# Idiopathic normal pressure hydrocephalus: survey on current diagnostic and therapeutic procedures in clinical practice in Germany

**DOI:** 10.1007/s00701-024-06354-x

**Published:** 2024-11-25

**Authors:** Fadi Al-Tarawni, Arif Abdulbaki, Manolis Polemikos, Jan Kaminsky, Hans A. Trost, Johannes Woitzik, Joachim K. Krauss

**Affiliations:** 1 Department of Neurosurgery, University Medical Center Hamburg-Eppendorf, Hamburg, Germany; 2 Department of Neurosurgery Hannover Medical School, MHH, Carl-Neuberg-Str. 1, 30625 Hannover, Germany; 3 Commission of Technical Standards and Norms, German Society of Neurosurgery, Hannover, Germany; 4 Department of Neurosurgery, Sankt Gertrauden-Krankenhaus, Berlin, Germany; 5 Department of Neurosurgery, University Hospital, Evangelisches Krankenhaus, Oldenburg, Germany

**Keywords:** Cognition, Gait, Idiopathic normal pressure hydrocephalus, Shunt surgery, Spinal tap test, Ventriculoperitoneal shunt

## Abstract

**Objective:**

Cerebrospinal fluid (CSF) shunting has become the standard treatment for idiopathic normal pressure hydrocephalus (NPH). Nevertheless, there is still disagreement on diagnostic criteria for selecting patients for surgery and optimal shunt management. The primary aim of the present study was to provide an update on the status of best practice, the use of different diagnostic algorithms and therapeutic management of idiopathic NPH in an European country.

**Methods:**

A standardized questionnaire with sections on the assessment of clinical symptoms and signs of NPH, diagnostic work-up, therapeutic decision making, and operative techniques was sent to 135 neurosurgical clinics in Germany that regularly perform shunt surgeries.

**Results:**

Overall, responses were received from 114/135 (84.4%) clinics. Most responders considered gait disturbance to be the hallmark clinical sign of idiopathic NPH (96%). A lumbar tap test was utilized always/ mostly by 97 centers (86%). In 43% of the centers, 30–40 ml CSF were removed with the spinal tap test. Spinal dynamic CSF studies were used by 12 centers only occasionally, and only by 1 center always for diagnostic purposes. Ventriculo-peritoneal shunting was the most frequent type of CSF diversion (> 90%). Pressure-controlled valves were used by the majority of units (95%) Overall 102 centers (93%) always/mostly used adjustable valves, and antisiphon devices were used always/ mostly in 50% of units.

**Conclusion:**

The present survey demonstrates that there has been a remarkable change of practice and opinions on the diagnosis and treatment of idiopathic NPH over the past two decades in Germany. Remarkably, variabilities in practice among different centers are less common than previously and recommendations according to scientific publications and guidelines have been implemented more readily.

## Introduction

While the concept of idiopathic normal pressure hydrocephalus (NPH) has been introduced in the 1960s [1, 55], it has received more attention over the past two decades [6]. It is currently accepted that the clinical entity is defined primarily by a gait disorder most often accompanied by cognitive impairment and urinary dysfunction [6, 38, 46]. Nevertheless, the nosology of idiopathic NPH and the differentiation from, respectively its coexistence with, other disorders has created much academic debate [3, 13, 28, 35]. There are several gaps and controversies, which still need to be addressed [15, 16, 53].

Despite the publication of several international and national guidelines for the diagnosis and treatment of idiopathic NPH [20, 22, 45], there is still a wide variety in both opinion and clinical practice on several aspects including diagnostic criteria, the use of diagnostic tests, confirmation of diagnosis, timing of surgery, and selecting the most suitable shunt systems for cerebrospinal fluid (CSF) diversion [19]. Despite the rapidly increasing number of publications and guidelines, the current clinical practice of diagnosing and treating idiopathic NPH remains largely unknown.

When Vanneste and van Acker published their survey *Normal pressure hydrocephalus: did publications alter management?* in 1990, they indicated that there was a large gap between recommendations given in publications and their implementation in clinical practice [54]. In contrast to their study, we noted that according to a contemporary survey in the late 1990s new diagnostic and therapeutic concepts on idiopathic NPH had penetrated daily routine to a certain extent although there were still marked discrepancies [27]. The purpose of the present study was to give an update on the status of best practice, the use of different diagnostic algorithms, and the therapeutic management of idiopathic NPH according to a nationwide survey in neurosurgery departments in Germany. Further, we discuss the findings both with regard to changes in practice according to our previous survey and current recommendations.

## Material and methods

A standardized questionnaire was developed by the Commission of Technical Standards and Norms of the German Society of Neurosurgery structured within three main sections with a total of 34 questions. The design of the questionnaire was based on a previous questionnaire on the diagnosis and treatment of idiopathic NPH [27], and another standardized survey on the practice of external ventricular drainage conducted by the commission [9]. The three sections covered questions on the estimated frequency of idiopathic NPH and the volume of shunt surgery (6 questions), diagnostic algorithms and decision-making processes (13 questions), and treatment including postoperative management (14 questions) (see English translation in supplementary material). Time needed to complete the questionnaire was 30—40 min. The majority of questions requested numerical numbers or estimated percentages, sometimes with multiple choices, and few questions asked for open comments. The questionnaire was sent to the head of the department along with a letter explaining the purpose of the survey.

Out of the 158 neurosurgical departments or divisions listed in the directory of the German Society of Neurosurgery, a total of 135 were selected for the mailing of the questionnaire. Those 23 units which were excluded were not involved in the diagnosis or treatment of idiopathic NPH (focusing on subspecialties such as spine surgery), or there was a transition of department leadership. Questionnaires were sent out first in May 2020, and two reminders were issued. The last questionnaires were returned in late 2021.

The data were tabulated and analyzed in an Excel spreadsheet. Note, that not every question was answered by each respondent, and that percentages refer to the number of responses received for a specific question. Statistical analysis was conducted using SPSS 23 (version 23.0, SPSS, Inc. Chicago IL). Descriptive statistics were presented either as counts or percentages of the parameters. The Kruskal–Wallis nonparametric test was employed to evaluate and examine disparities in the collected data for statistical significance. The significance level was set at p = 0.05.

## Results

A total number of 114 questionnaires from the 135 neurosurgical units which had been contacted were returned, representing 84.4% of the targeted clinics. There were 34 responses from primarily academic centers, and 80 from others. The names of the respondents who completed the survey and the cities where they come from are listed in the Acknowledgements.

### Estimated frequency of idiopathic NPH and volume of shunt surgery

According to the survey an estimated number of a total of 4173 patients with a suspected diagnosis of idiopathic NPH were seen over a year (range, 6—200; median 25 patients per center). The majority of patients were first seen as outpatients (median 80%). Among those, about 80% were subsequently admitted as inpatients for additional examinations and treatment. It was estimated that the diagnosis was finally confirmed in about 70% of patients (range, 10%—100%), corresponding to an estimate of a total number of 2336 patients. While it was indicated that a total of 7705 patients had shunt surgery for any type of hydrocephalus (range, 2—250; mean 50 operations per center over a year), the estimated proportion of patients with idiopathic NPH was 25%, corresponding to a number of 1926 patients.

### Diagnostic algorithms and decision-making

#### Responsibility for decision-making for shunt surgery

The decision to proceed with shunt surgery was always/ mostly made by neurosurgeons alone in 72 units, by neurologists alone in 4 units, and by both disciplines in 30 units. Diagnostic evaluations were performed in 73 units (65%) either in the neurosurgery or the neurology department, in 31 units (27%) only in the neurosurgery department, and in 9 units (8%) only in the neurology department.

#### Clinical signs and symptoms

The majority of units considered gait disturbance to be the most important sign of idiopathic NPH (96%), whereas only few indicated dementia or urinary problems as most important (2%, respectively). A diagnosis of idiopathic NPH was considered in patients with a gait disturbance as the only clinical sign in 74% of the responding units, while the remaining 26% indicated that they required the presence of additional signs or symptoms (in case of appropriate imaging).

#### Diagnostic imaging findings

The Evans index was employed for imaging objectification by 61% of units, and the callosal angle was utilized by 59%. Both the Evans index and the callosal angle were utilized by 47%. Conversely, neither of these measures were employed in 26%. In an estimated mean of 60% of patients subjected to shunt surgery flattened sulci over the convexity were present, while the estimate for enlarged sulci was 10%. The DESH (disproportionately enlarged subarachnoid space hydrocephalus) phenomenon was estimated to be present in about 60% of patients.

#### Overview of diagnostic studies

A variety of diagnostic studies was used in the routine preoperative assessment of idiopathic NPH among the 113/ 114 neurosurgical units which provided information on this topic. An overview on the estimated frequency of the different investigations used is shown in Table 1.

**Table 1:** Overview on the frequency of diagnostic studies used in routine preoperative assessment of idiopathic NPH in a total of 113/114 responding neurosurgical units

		Always	Mostly	In about 50% of the patients	Sometimes	Never	No answer
1	CT	48	27	10	12	1	15
2	Single photon emission computed tomography (SPECT)	0	0	0	9	92	12
3	Positron emission tomography (PET)	0	0	0	8	95	10
4	MRI (standard, morphology)	49	45	7	11	1	0
5	MRI (CSF flow studies)	5	12	4	42	40	10
6	Lumbar CSF tap test	79	18	1	14	0	1
7	Lumbar CSF drainage	11	13	9	59	19	2
8	Ventricular CSF removal (following ICP monitoring)	1	2	0	27	78	5
9	Neurophysiological studies	6	14	6	50	32	5
10	Transcranial Doppler	0	2	0	25	80	6
11	Continuous ICP monitoring	2	2	1	25	80	4
	Epidural	0	0	0	3	56	54
	Subdural	0	0	0	4	54	55
	Intraventricular	2	2	0	17	52	40
12	Intraventricular bolus injection	0	0	0	1	102	10
	PVI	0	1	0	1	68	43
	Compliance	1	1	0	1	70	40
13	Lumbar infusion test	1	0	0	12	95	5
14	Lumboventricular perfusion	0	0	0	0	109	4

*CSF* Cerebrospinal fluid, *ICP* Intracranial pressure, *PVI* Pressure volume index

Conventional magnetic resonance imaging (MRI) or computed tomography (CT) scans were deemed essential in all units, with 19 respondents mandating the use of both modalities. MRI CSF flow studies were used always/ mostly in 17 centers, whereas they were never used in 40 units.

Single photon emission computed tomography (SPECT) and positron emission tomography (PET) imaging were rarely performed in clinical routine with 9 units employing occasionally SPECT, and 8 units occasionally PET.

A lumbar tap test was performed always/ mostly in the majority of neurosurgical units (86%). There was no unit which indicated that a lumbar tap test was never used. While 79 units always performed a tap test (70%), a probatory lumbar drain was placed always in 11 units (10%) for diagnostic purposes. CSF removal via a ventricular drain following intracranial pressure (ICP) measurements was performed always/ mostly in 3 units.

Neurophysiological studies were used in 20 units always/ mostly, conversely, the majority of respondents reported infrequent utilization. Transcranial Doppler was utilized mostly in 2 units, whereas this was never the case in 80 units.

ICP monitoring was used always/ mostly in only 4 units, while this was never the case in 80 units. Dynamic investigations of CSF pressure homeostasis, including intraventricular bolus injection, lumbar infusion tests, and lumboventricular perfusion tests, were performed always/ mostly in only 4 units. While intraventricular CSF dynamics were investigated occasionally in 17 units, spinal CSF dynamics were investigated occasionally in 12 units, and always in only 1 unit.

#### Lumbar CSF removal

The lumbar tap test was considered the most relevant diagnostic procedure, followed by probatory lumbar drainage, and MR imaging. The majority of respondents (84%) also indicated that they based their decision to recommend shunt surgery primarily on the result of the lumbar tap test. The volume of CSF which was removed with the spinal tap test ranged between 20 and 50 ml (20 ml was removed by 9% of the respondents, 30 ml by 39%, 40 ml by 43%, and 50 ml by 9%). When asked for signs and symptoms which were evaluated before and after the spinal tap test, all units except one (99%) indicated that they assessed patients´ gait, 78% assessed cognitive function, and 47% the whole triad. Standardized evaluations were performed in 77/ 114 units (68%). The Mini Mental Score examination was utilized in 83 units (72%), and the MoCa-Test in 23 units (20%). The majority of units considered also the global impression of change after the tap test (81%), patient´s subjective impression (89%), and the judgement of third persons such as relatives or nurses (97%).

### Treatment including postoperative management

#### Shunt surgery

Ventriculo-peritoneal shunt insertion was the most frequent type of CSF diversion (> 90%), followed by ventriculo-atrial shunting (about 5%). Lumbo-peritoneal CSF diversion was performed on a more regular basis only in 2 units.

#### Antibiotics

All units administered intravenous antibiotics prior to skin incision. Postoperative antibiotics were given in 6 units.

#### Insertion of the ventricular catheter

The shunt catheter was inserted into the frontal horn always/ mostly in the majority of units using a free-hand approach (95%). Ultrasound guidance was used always/ mostly in 4 units, navigation in 1 unit, while guiding instruments such as the Thomale guide were used only occasionally in 7 units. Three units indicated that they never inserted the ventricular catheter with a free-hand approach.

#### Valve types

Adjustable valves were inserted always/ mostly in 93% of the units, while no center indicated that they never used such valves. Valve settings were changed during the postoperative course on a more regular basis in 36% of units. Antisiphon devices were used always/ mostly in 50% of units. Adjustable antisiphon devices, however, were employed always/ mostly only rarely (5%), and were never used in idiopathic NPH in 52% of units.

#### Alternative treatment options

Apart from classical CSF diversion other treatment options were used only exceptionally. Ventriculocisternostomy was utilized more frequently in 2 units, and ventricular-sinus shunting occasionally in 3 units.

#### Evaluation of clinical outcome

The therapeutic efficacy was based mainly on patient feedback (100%). Outcome scales such as the Kiefer score were utilized always/ mostly in 12 units.

#### Estimates of postoperative outcome and complications

Pooling the mean estimates from all units indicated that the overall improvement after CSF shunting was judged as follows: nil in 10% of patients, mild in 14%, moderate in 26%, and marked in 50%. The pooled mean estimates for complications were: intraoperative complications (2%), postoperative complications such as thrombosis or pneumonia (3%), postoperative infections (3%), underdrainage (13%), overdrainage (10%), subdural hygroma (7%), and subdural hematoma (4%). Patients were seen postoperatively for more than 1 year in 75% of units, and for more than 3 years in 47%.

## Discussion

The present survey demonstrates that there has been a remarkable change of opinions on the diagnosis and treatment of idiopathic NPH over the past two decades in clinical practice in Germany where most of neurosurgeons are organized under the umbrella of a national neurosurgical society [27]. Considering the relatively high number of units which responded to the survey, it can be assumed that the results of the study are representative for what actually reflects daily routine and how new methods have been implemented while several other concepts have been abandoned. Nevertheless, there is still a wide variability among different neurosurgical units, although this variability has become much smaller when compared to previous surveys [27, 54].

As outlined previously, any survey provides valid data only for a limited period of time, and in addition to demonstrating the status quo, it also shows periods of transition in changing clinical practices. In contrast to the early publication of Vanneste and van Acker [54], it is obvious now that novel scientific data are much more rapidly installed and their impact on both diagnosis and treatment is much higher than this was the case before [55]. In particular with that regard idiopathic NPH now has become a much less *enigmatic disease* [16].

There is still limited data on the epidemiology of idiopathic NPH, and divergent prevalence and incidence rates have been reported [10, 23, 32, 50]. While initially it has been thought that idiopathic NPH would rather be a rare occurrence, more recent studies have reported prevalence rates in persons aged 65 years or older varying between 0.5 and 8.9% [10, 23, 32, 50], depending mainly on differing diagnostic criteria and methodological issues. According to a population-based study in Gothenburg, the prevalence of idiopathic NPH was 1.5% in a study cohort of 791 individuals aged 70 years [10]. According to our present survey, about 4,170 patients with a suspected diagnosis of idiopathic NPH were seen over a period of a year in neurosurgical departments across Germany, with a number of about 2,300 patients in whom the diagnosis was confirmed. Considering the population of 83,800,000 in 2022 in Germany, the estimated annual incidence of idiopathic NPH would be 2.8 cases pro 100,000 inhabitants. In addition, the estimated annual incidence of shunt surgery for NPH would be 2.3 cases per 100,000 inhabitants. Again, the true incidence of NPH would be expected to be much higher since according to our methodology only patients with more marked symptoms having appropriate medical access would be accounted for. Notably the incidence of idiopathic NPH in our previous study from 2004 was estimated to be at 1.8 cases per 100,000 inhabitants [27], which would mean a 1.5-fold increase in the overall incidence which might be related to various factors including increased awareness of the disease entity, more frequent use of imaging studies, and an increasingly aging population.

It appears that the majority of diagnostic evaluations in patients with idiopathic NPH are performed in neurosurgical units rather than in neurology departments. These figures are comparable to the numbers which were obtained two decades ago, however, it is remarkable that there is a more close collaboration now in about 25% of centers. These numbers appear to coincide with similar figures worldwide indicating that patients with idiopathic NPH are either being seen by neurologists or neurosurgeons, however, rarely by specialists in gerontology.

Surprisingly and clearly reflecting the impact of recent publications is the fact that about 96% of responders now consider gait disturbance to be the most important sign of idiopathic NPH (as compared to 61% in the previous survey) [44, 48, 58]. Also, the high number of responders considering that gait disturbance might be the only clinical sign of idiopathic NPH (74% of the responders) indicates, that there has been a more radical paradigmatic change in the clinical diagnosis of idiopathic NPH and that the clinical entity according to contemporary opinion is more considered a *treatable gait disorder* than a *treatable form of dementia* [6, 44].

Diagnostic studies have been the focus of several national and international guidelines published over the past decades [20, 22, 42, 45]. In Germany, there have been regular updates of a common guideline of the German societies of neurology and neurosurgery about every five years [45]. The shift of diagnostic recommendations now is also reflected in our current survey showing that the lumbar tap test has become the most relevant diagnostic procedure while several other methods are being performed only in few selected centers and others have been abandoned [19]. The choice of imaging studies to document hydrocephalus and associated features in idiopathic NPH has shifted more towards MRI [17, 49]. While MRI CSF flow studies were propagated in the literature according to several publications [5, 26, 29], they are only more rarely considered in clinical practice according to our present survey, and less centers pay attention to such findings in comparison to 20 years ago [27]. Also, PET and SPECT investigations are performed more rarely nowadays than before, and they are not used as routine algorithms in any department, despite they might inform about changes in periventricular blood flow and hemodynamics which have been thought to be relevant in the pathophysiology of idiopathic NPH and which might also have an impact on the prediction of the improvement after shunt surgery.

As recommended in the national guideline [45], more attention is now being paid to the objectivation of imaging findings. This is exemplified by the fact that the DESH phenomenon was estimated to be present in about 60% of patients, (but also that methods such as the Evans index and the callosal angle were utilized on a routine basis in the majority of centers [34, 37].

The lumbar tap test is considered the most relevant diagnostic procedure to confirm the suspected diagnosis of idiopathic NPH [15, 56, 57]. Compared to our earlier survey, the present results show that there is much less variation in the amount of CSF being removed and that a total of 83% would either remove 30 or 40 ml for diagnostic assessment. Interestingly, several units also use lumbar drainage for confirmation of NPH diagnosis [12, 18, 30, 43]. Another relevant finding is that practically all units would now evaluate gait as the primary symptom after CSF removal [33]. With regard to the more uniform performance of the spinal tap test and the higher frequency of standard evaluations thereafter, it may be expected that overall the tap test might become more reliable allowing also a better comparison of studies from different centers.

Remarkably, only few centers still perform ICP monitoring or dynamic investigations of CSF pressure homeostasis on a regular basis [7, 24, 36, 57]. This is in stark contrast to our previous survey but this also in contrast to guidelines published over the past few years. The reduced use of such methods might be related to a more practical approach when relying mainly on the results of CSF removal, however, also to the ongoing difficulties in interpreting the results of the more complex monitoring procedures which also require longer periods of patient hospitalization.

While CSF diversion via shunt surgery is still the most accepted treatment for idiopathic NPH and there has been little change with that regard, there have been several remarkable changes concerning both techniques and technology [2, 4]. The placement of ventriculo-peritoneal shunts now has become common practice, while ventriculo-atrial shunts are placed at a much lower frequency at about 5% with some variations according to local practices. In contrast to published studies on the efficacy of lumboperitoneal shunting and the frequent mentioning of this method in several guidelines [21, 41], our survey shows that in daily practice this method is almost never applied in the treatment of idiopathic NPH in Germany. Notably, there has been a remarkable change in the choice of valve types which are used for shunt surgery of idiopathic NPH [8, 31, 47]. While according to our previous study the majority of centers had not used adjustable valves, such valves are inserted nowadays always or mostly in 93% of the responding centers, and even more remarkably anti-siphon devices are used always or mostly in about half of the centers. The use of such valve types might also be related to the fact that both overdrainage and underdrainage is estimated to occur less frequently than before. With that regard it is particularly relevant, that overall the use of new shunt technologies has resulted in reduction of subdural fluid collections and in revision surgeries when compared to fixed valves used earlier in several international studies [14].

Our survey also confirms that alternative treatment options apart from shunt surgery have little significance in the treatment of idiopathic NPH. While the concept of CSF shunting to the superior sagittal sinus, although attractive from a practical point of view, has been practically abandoned over the past view years [52], there is still a controversial discussion about the possible value of endoscopic ventriculocisternostomy in idiopathic NPH [25, 39].

Estimates both on positive outcome and complications appear to be more optimistic than previously which might reflect better patient selection by more uniform and improved diagnostic tests, and the application of more standardized techniques for shunt insertion and shunt technology [11, 51]. Certainly, there is still a lack of long-term outcome studies [40] and, in particular, differential diagnoses of idiopathic NPH with regard to neurodegenerative disorders such as progressive supranuclear palsy deserve further consideration [15].

In conclusion, our study demonstrates several changes in practice in the diagnosis and treatment in a large European country. Remarkably, variabilities in practice are less among different centers than in earlier times and recommendations according to scientific publications and guidelines are implemented more readily. Although our findings cannot be extrapolated easily to other geographical regions, they clearly show that the clinical picture of idiopathic NPH is now defined much better and treatment strategies have become safer and more easily implemented.

## Data Availability

Data sets are available upon reasonable request.

## Abbreviations

CSF: Cerebrospinal fluid
CT: Computed tomography
DESH: Disproportional enlarged subarachnoid space hydrocephalus
ICP: Intracranial pressure
MRI: Magnetic resonance imaging
NPH: Normal pressure hydrocephalus
PET: Positron emission tomography
SPECT: Single photon emission computed tomography
